# Aquatic suspended particulate matter as source of eDNA for fish metabarcoding

**DOI:** 10.1038/s41598-020-71238-w

**Published:** 2020-09-01

**Authors:** Cecilia Díaz, Franziska-Frederike Wege, Cuong Q. Tang, Alexandra Crampton-Platt, Heinz Rüdel, Elke Eilebrecht, Jan Koschorreck

**Affiliations:** 1 Department of Ecotoxicology, Fraunhofer IME, Auf dem Aberg 1, 57392 Schmallenberg, Germany; 2 Nature Metrics, CABI Site, Bakeham Lane, Egham, Surrey UK; 3 Federal Environment Agency (UBA), Bismarckplatz 1, 14193 Berlin, Germany

**Keywords:** Biological techniques, Ecology

## Abstract

The use of environmental DNA (eDNA) for monitoring aquatic macrofauna allows the non-invasive species determination and measurement of their DNA abundance and typically involves the analysis of eDNA captured from water samples. In this proof-of-concept study, we focused on the novel use of eDNA extracted from archived suspended particulate matter (SPM) for identifying fish species using metabarcoding, which benefits from the prospect of retrospective monitoring and also analysis of fish communities through time. We used archived SPM samples of the German Environmental Specimen Bank (ESB), which were collected using sedimentation traps from different riverine points in Germany. Environmental DNA was extracted from nine SPM samples differing in location, organic content, and porosity (among other factors) using four different methods for the isolation of high-quality DNA. Application of the PowerSoil DNA Isolation Kit with an overnight incubation in lysis buffer, resulted in DNA extraction with the highest purity and eDNA metabarcoding of these eDNA fragments was used to detect a total of 29 fish taxa among the analyzed samples. Here we demonstrated for the first time that SPM is a promising source of eDNA for metabarcoding analysis, which could provide valuable retrospective information (when using archived SPM) for fish monitoring, complementing the currently used approaches.

## Introduction

Suspended particulate matter (SPM) is one of the fundamental elements in aquatic ecosystems (in addition to the water phase and the sediment) and, because it can act as a source and transport mechanism for aquatic particles, is useful for assessing the contamination of surface water^1^. As such, SPM monitoring is an important consideration under the Water Framework Directive 2000/60/EC (WFD)^2^ and is used in European regulatory chemical monitoring programs (e.g. in Germany^3^ and Netherlands^4^). Among the other quality elements monitored under the WFD is the state of the aquatic macrofauna (e.g. fish communities), which provide a good measure for the biological and chemical status of the water bodies. SPM monitoring for this context is yet to be explored.

Traditionally, the response of biotic communities to human-induced stressors are assessed using taxonomic approaches (e.g. electrofishing), but these are typically limited by their scalability, restricted temporal and spatial resolution, invasiveness, and the high level of expertise required to identify the species^5^. Moreover, rare taxa (e.g. invasive species—*Neogobius melanostomus*, or migratory species—*Salmo salar*, *Anguilla anguilla, Alosa alosa*) are especially difficult to monitor because they require an intensive survey effort. DNA-based tools can overcome many of the above-mentioned problems and could complement traditional approaches and biomonitoring strategies^6^.

Recently, the use of environmental DNA (eDNA), which is the trace amounts of DNA discharged by organisms via excretion of urine, feces, body cells, eggs, etc., enables the non-invasive detection of species and their approximate relative quantification^6–9^. Whole fish communities can be characterized from eDNA using high throughput sequencing or specific amplicons—a process known as eDNA metabarcoding^10–14^. Fish eDNA metabarcoding is now an emerging tool that has been adopted in hundreds of primary studies and for the most part has relied on either eDNA capture from the water phase (e.g. by filtering water) or the from the sediment. eDNA captured by filtering water is cheap and relatively easily sampled and provides a snapshot of the diversity at the sampling point^11,15^, while sediment eDNA benefits from high DNA yields owing to the continuous sedimentation of eDNA and might be useful for long-term site occupancy^15–18^.

An as yet unexplored component of the aquatic environment for the eDNA monitoring is the SPM, which, if it behaves similarly to sediment, could benefit from high DNA yields, while also offering a contemporary snapshot of the diversity. Moreover, archival SPM could be used to explore temporal diversity variation. The German Environmental Specimen Bank (ESB) has archived annual composite samples of SPM from 13 river sites since 2005^19^. These SPM samples could represent a valuable source of material for retrospective seasonal and temporal fish community monitoring. In this proof-of-concept study, we aim to demonstrate, that eDNA extracted from archived SPM samples can be used for identifying fish species using metabarcoding of the 12S rRNA gene. To our knowledge, this represents the first evaluation of SPM as a source material for eDNA-based evaluation of aquatic macrofauna.

## Results

Four different protocols for the isolation of high-quality DNA from SPM samples were compared: First-Magnetic Forensic Kit (GEN-IAL), Nucleo Spin Soil Kit (Macherey–Nagel), DNeasy PowerSoil Kit (QIAGEN) and a modification of the protocol (overnight incubation in lysis buffer) of Power Soil Kit. The spectrophotometric analysis showed that all kits tested yielded enough amount of DNA. The First-Magnetic Forensic Kit showed highest amount of DNA extracted (factoring in sample weight) but suffered from having the least pure DNA (Table 1; ~ 1.8 was considered as pure DNA (spectrometric ratio 260/280 nm) as per^20^). The modified PowerSoil protocol resulted in the best balance between DNA yield and purity of DNA and was therefore chosen as the method to extract DNA from the archival SPM samples, which were subsequently analyzed by eDNA metabarcoding.

**Table 1:** Concentration and purity of the DNA extraction method tested, determined spectrophotometrically. For each method, 2 SPM samples were extracted.

Extraction kit	Sample	SPM amount (mg)	DNA concentration [ng/μl]	DNA concentration [µg/g]	Purity [260/280]
First-magnetic forensic kit	SPM 1	47.4	50.53	50.53	1.43
	SPM 2	60.7	40.90	40.90	1.44
Nucleo spin soil kit	SPM 1	313.15	134.90	21.79	1.83
	SPM 2	263.35	83.40	15.80	1.80
DNeasy PowerSoil kit	SPM 1	240.0	44.15	17.87	1.73
	SPM 2	245.8	43.40	17.66	1.73
DNeasy PowerSoil kit^a^	SPM 1	250	75.65	30.26	1.89
	SPM 2	250	47.05	18.82	1.79

^a^Modified original protocol: extended (overnight) incubation in the extraction buffer.

The DNA from nine SPM samples were extracted and the concentration and purity is summarized in Table 2. PCR reactions, targeting a 170 bp 12S rRNA region designed for fish monitoring^11^, were consistently successful for the samples of the nine sites analyzed. Electrophoresis bands were strong and of the expected size for each of the 12 PCR replicates conducted per sample. High throughput sequences of these indexed PCR products yielded a total of 1,221,036 raw sequences from which 886,820 quality filtered (USEARCH) and 137,939 dereplicated sequences were generated. After removal of sequences indicative of PCR and sequencing error, a total of 717,011 high- quality sequences were retained in the final taxon-by-sample table.

**Table 2:** DNA concentration and purity of the SPM samples.

Sample location	DNA concentration [ng/μl]	Purity [ratio 260/280 nm]
Koblenz	44.9	1.83
	40.1	1.76
	36.8	1.82
Weil	38.7	1.79
	39.4	1.81
	31.9	1.79
Bimmen	47.8	1.81
	39.3	1.78
	45.3	1.76
Ulm	59.0	1.77
	57.7	1.78
	57.7	1.77
Kelheim	64.0	1.78
	58.4	1.77
	54.6	1.81
Prossen	62.9	1.81
	60.8	1.81
	42.5	1.83
Dessau	51.6	1.78
	53.2	1.84
	55.6	1.82
Blankenese	29.4	1.77
	28.9	1.78
	24.7	1.77
Güdingen	47.5	1.77
	53.3	1.78
	62.4	1.77

Samples were extracted in triplicate using the DNeasy PowerSoil kit with an extended incubation with the extraction buffer.

**Figure 1:**
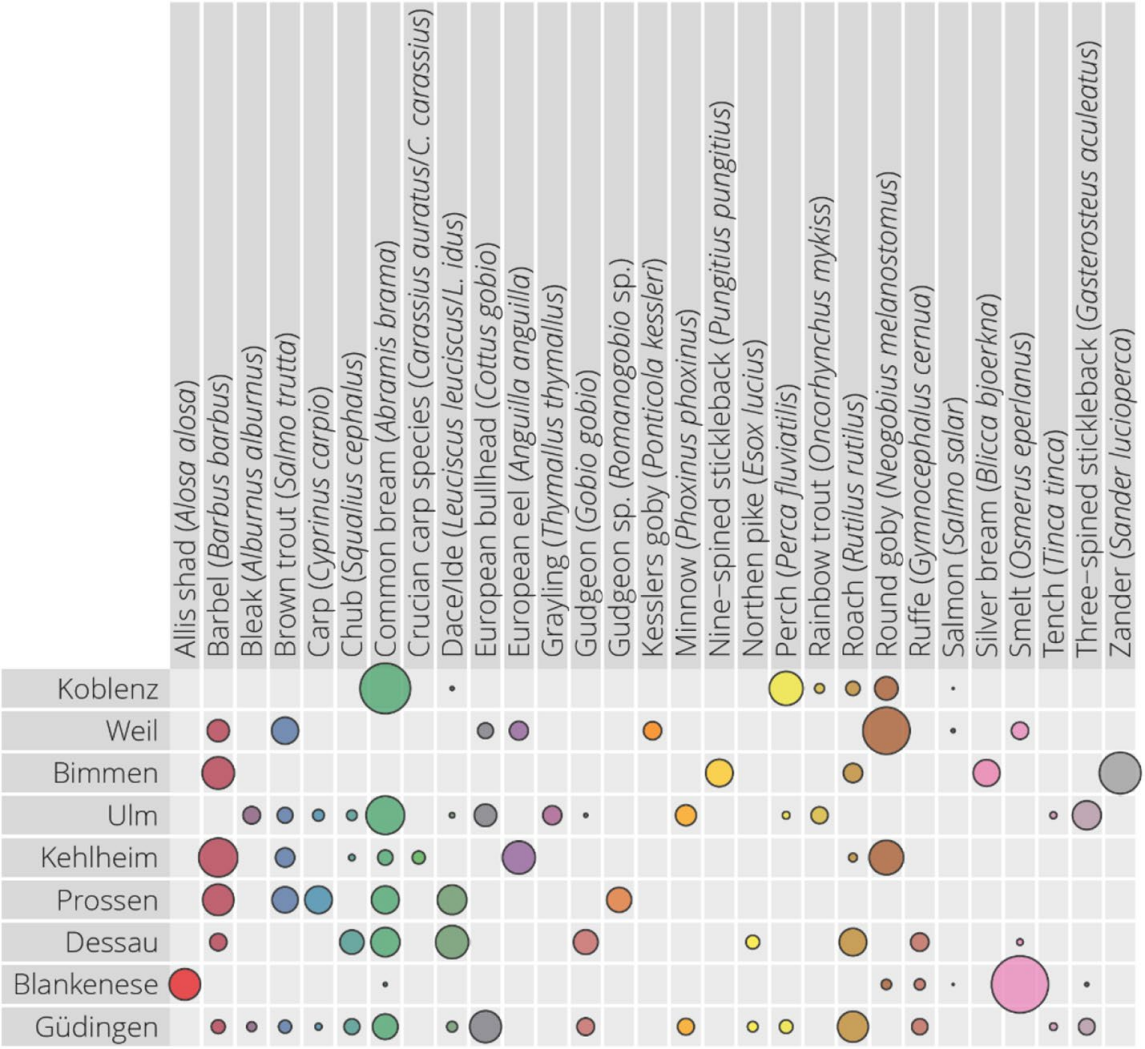
Proportion of the sequencing output allocated to the different species.

A total of 29 taxa were detected across the nine sites sampled from which 27 were identified to species level. The fish species belong to 11 orders: Anguilliformes, Beloniformes, Clupeiformes, Cypriniformes, Esociformes, Gasterosteiformes. Gobiiformes, Osmeriformes, Perciformes, Salmoniformes, and Scorpaeniformes), and 11 families (Adrianichtyiadae, Anguillidae, Clupeidae, Cottidae, Cyprinidae, Esocidae, Gasterosteidae, Gobiidae, Osmeridae, Percidae, and Salmonidae). The relative proportion of the fish sequences found in each of the samples is shown in Fig. 1. The species richness ranged from 5 (Bimmen) to 17 (Güdingen). The diversity richness is summarized in Table 3. The lower number of species detected in Bimmen correlated with the lower amount of sequences returned for this sampling owing to loss of sequences from bacterial non-target amplification as indicated by a double banding at the PCR stage. The number of sequences for these samples was on average 44 percent of the other samples. Common bream (*Abramis brama*), which accounted for 13% of the total sequence reads, was the most abundant in terms of sequences. Among the most commonly detected species were the common bream, barbel (*Barbus barbus*), and roach (*Rutilus rutilus*), which were detected in 9, 8, and 5 of the 9 samples, respectively. All the species found have been already reported for German rivers (See Supplementary Information).

**Table 3:** Diversity richness among the samples.

Sample location	Order	Family	Genus	Taxa (IDed* to species)
Koblenz	4	4	8	8 (7)
Weil	7	7	8	9 (9)
Bimmen	3	3	5	5 (5)
Ulm	5	5	14	14 (13)
Kelheim	4	4	8	8 (7)
Prossen	2	2	6	6 (4)
Dessau	4	4	10	10 (9)
Blankenese	8	8	8	8 (8)
Güdingen	6	6	17	17 (16)

^a^IDed = identified/assigned to species level.

## Discussion

As we hypothesized, the applicability of using SPM as source for fish eDNA metabarcoding has been confirmed and used for first time in this study. Fish species were found in all samples, irrespective of the location or characteristics of the SPM sampled.

Comparing the different extraction methods used, the eDNA extracted from SPM samples using a modified protocol of the DNeasy PowerSoil Kit, presented the highest purity (260/280 nm ratio) in combination with high DNA concentration, therefore it was the method selected for metabarcoding the eDNA extracted from the nine sampling sites. The isolation method was chosen due to its simplicity and scalability to perform a high number of extractions. However all tested methods resulted in high DNA concentration, making them suitable for metabarcoding, even if post extraction cleanup would have been needed (e.g. the Magnetic Forensic kit showed lower purity 1.44 (260/280 nm ratio)).

While eDNA-based fish monitoring from filtered water samples has been widely used and described and has cheap setup costs, it provides only a snapshot of the diversity at the sampling point, while continuous integration and eDNA settling in time-integrative sampled SPM would provide a better reflection of long-term site occupancy^15–18^. On the other hand, eDNA extraction from water samples using filters are laborious and extractions yields are low. The process of particles sinking or binding of eDNA (or residues of, e.g. fish tissue, feces or shales containing eDNA) to organic or mineral particles in SPM^18^ may result in a progressive accumulation of eDNA in the SPM. This statement was confirmed in our study. The results showed that using one SPM sample yielded higher DNA amounts per extraction (400–2,500 ng) than what is reported for eDNA extracted from an individual water sample using filters (30–560 ng)^18,21–25^. Here a small amount of SPM (~ 250 mg) is sufficient to extract high amounts of eDNA, which is of particular importance for the detection of rare fish species, where the concentration of their DNA is expected to be low. For example, *Salmo salar* which is classified as endangered in German rivers^26^, was detected in the Koblenz, Weil, and Blankenese SPM samples. Another main advantage of using SPM (in particular archived in the ESB), is that it is possible to retrieve and reanalyze the source material, allowing repeats and other complementary analyses e.g. chemical analysis to determine the presence of contaminants or stressors responsible for changes in fish populations. This kind of repeat analysis are not possible with filtered water samples, unless multiple samples are taken in parallel or the water itself is retained, both costly options.

Here, eDNA metabarcoding of the 9 riverine sites detected a total of 29 fish species. Most taxa found belong to commonly detected species in large rivers in Germany. For example, *Abramis brama*, *Rutilus rutilus*, *Barbus barbus*, *Squalius cephalus*, and *Perca fluviatilis* and are largely overlapping with the regulatory monitoring data from the Water Framework Directive (WFD)^27^. This coherence of fish species identified from eDNA extracted from SPM with the commonly detected fish species demonstrated the suitability of this approach. However, the number of fish species found in the ESB samples is similar or lower to what was found using traditional fish monitoring techniques, e.g. electro- and netfishing under the WFD^27^. For example ,with regard to monitoring sites in Germany between 27 and 57 fish species have been detected in 2012 and 2013 along the Rhine^28^, between 19 and 24 fish taxa were counted in 2007 at four sites of the river Elbe and between 27 and 29 fish species were detected at three sites of the Danube^29^. However, it needs to be considered that the number of WFD surveillance monitoring sites is much higher than the ESB sampling sites investigated in this study.

The fish community analysis also evidenced the presence of two contaminant species: *Danio rerio* and *Oryzias latipes*. For this reason, the extractions from the 9 sampling sites were repeated retrieving new subsamples from SPM, and before sequencing the absence of contaminant species (e.g. *Danio rerio*) was validated using specific qPCR primers (See Supplementary information). The specie-specific qPCR and the metabarcoding results showed successful removal of exogenous lab- contaminant fish species (See Supplementary information). The detection of those reads in the first samples strongly suggests cross-contamination in the laboratory since *Danio rerio* is a specie that we used commonly in our facilities for other purposes. It is well known that the most serious pitfall of metabarcoding eDNA is the risk of contamination with exogenous DNA^30,31^.

At the stage of PCR during library preparation, several samples exhibited unspecific amplification (double banding), Prossen, Weil, Bimmen and Dessau, which might be indicative of bacterial amplification. This additional bacterial amplification might have resulted in less efficient fish-specific sequencing and in consequence, a lower number of species found in those samples (5–9 species found compared to 8–17 species found in the non-contaminated samples). However, the richness is not only attributable to the presence or absence of contamination but might be also inherent to the sample. Contamination of reagents with bacterial DNA, or contamination with exogenous DNA in the laboratory (e.g. *Danio rerio*), in combination with the bacteria inherent to the sample itself, is a major problem exacerbated by the highly sensitive nature of the PCR, in particular when using universal primers. Therefore, even minor presence of these species in the lab equipment (like pipettes, surfaces, etc.) might result in large non-target amplification. To avoid such risk, we performed decontamination procedures for laboratory spaces and equipment (with UV radiation) and physically separated pre- and post-PCR workspaces.

The results of this proof-of-concept study will open the door for the retrospective evaluation of SPM samples to study, for example, seasonal and temporal trends of invasive species. The present study can be regarded as a first step towards more comprehensive investigations using eDNA extracted from archived SPM of freshwater fauna, flora and microorganisms. The fish taxa detected in this study complement well with species sampled in fish monitoring with traditional methods, e.g. nets, fykes and electrofishing. However, to study the fish community of a particular sampling site and draw conclusions on differences among sites, further investigations and more stringent analyses are required. The definition of a methodology should include an eDNA extraction strategy considering, for example, SPM extraction volume, the number of replicate extractions, the number of independent sequencing analyses required vs pooling the extracted DNA, etc. In order to validate this proof-of-concept study, future work will focus on method optimization and comparisons with established monitoring approaches.

## Methods

### SPM sampling

**Figure 2:**
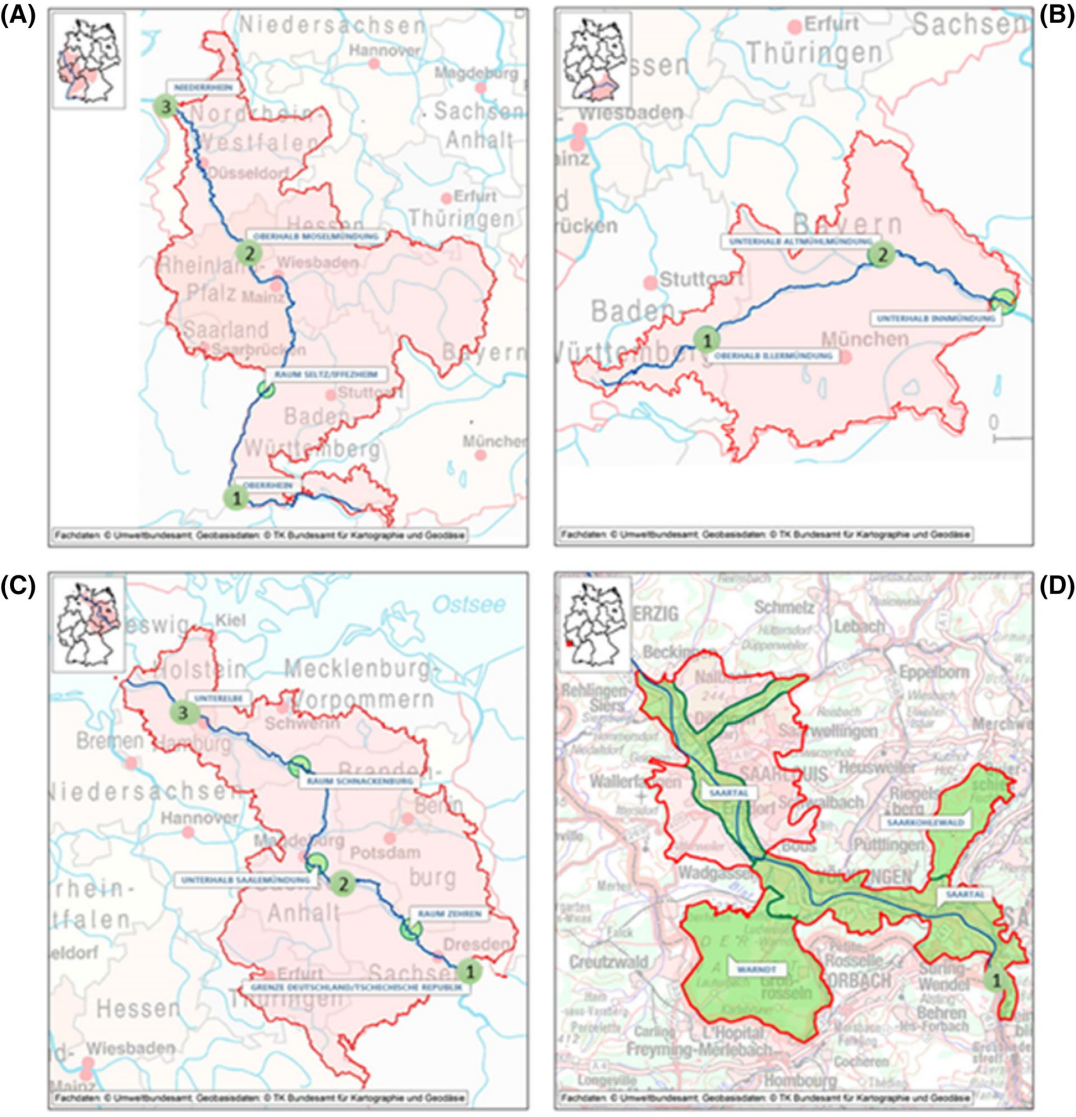
SPM riverine sampling locations. (**a**) Rhine river: 1) Weil, 2) Koblenz, 3) Bimmen; (**b**) Danube river: 1) Ulm, 2) Kelheim; (**c**) Elbe river and tributaries: 1) Prossen, 2) Dessau (Mulde river), 3) Blankenese; (**d**) Saar river: 1) Güdingen. Sampling sites maps were taken from: https://www.umweltprobenbank.de/en/documents/profiles/specimen_types/14940.

In order to validate the use of SPM as DNA source for metabarcoding analysis, subsamples from the 2016's annual SPM samples were retrieved from the cryo-archive of the German ESB which is operated on behalf of the German Environmental Agency (Umweltbundesamt, UBA). The sampling sites correspond to nine different riverine sites in Germany (Fig. 2), from the river Rhine (Weil and Koblenz), Danube (Ulm and Kelheim), Elbe and tributaries (Prossen, Dessau, and Blankenese) and Saar (Güdingen). The chosen sites represent SPM samples with different characteristics (e.g. organic matter content, porosity, granularity), and fish communities. The samples were collected according to the ESB guidelines for sampling and sample processing^19,32^. In brief, stainless steel sedimentation boxes are permanently installed in monitoring stations or directly in rivers, to collect SPM samples. Samples are gathered monthly, the supernatant water from these samples is removed and the remaining SPM sieved (≤ 2 mm), homogenized, and frozen on site. Frozen samples are transported to the ESB cryo-archive and stored in cryo-containers in the gas phase above liquid nitrogen (< − 150 °C). The samples in this study correspond to annual samples, in which at least 6 kg of the monthly samples are pooled and subsampled (200 subsamples of about 10 g dry weight). Each of the nine samples used for this study belong to one subsample. In case of repeats, a new subsample was retrieved and used.

### eDNA extraction from SPM

Three different extraction kits for the isolation of high-quality genomic DNA from SPM samples were tested: First-Magnetic Forensic Kit (GEN-IAL), NucleoSpin Soil Kit (MACHEREY-NAGEL), and the DNeasy PowerSoil Kit (QIAGEN). The extractions were performed according the manufacturer’s instructions. In addition we also tested a modified protocol for the PowerSoil kit, which included homogenization with a FastPrep (2 × 30 s), and an extended (overnight) incubation at 60 °C in a Thermomixer. All four DNA extraction methods were performed using 2 different SPM samples (called SPM 1 and SPM 2). The yield and purity of the DNA extracts was measured spectrophotometrically using a NanoDrop ND-2000 (ThermoScientific, USA).

The DNA extraction of the annual SPM samples for the high-throughput DNA analysis was performed in triplicate using the modified protocol of the DNeasy PowerSoil Kit. After extraction, yield and purity were measured using a NanoDrop ND-2000 (ThermoScientific, USA). The triplicate DNA extracts, from each SPM sample, were mixed and pooled to generate a unique sample for high throughput sequencing analysis.

### Metabarcoding and sequencing

Before and after each step, all benches were decontaminated with 10% commercial bleach followed by DNA wipes (Minerva Biolabs). Each step of the process had its own designated space, equipment, reagents and consumables. A hypervariable region of 12S rRNA was amplified via a two-step PCR process. In the first step, purified DNA was amplified with MiFish primers^11^ modified to correct a mismatch between the second base pair of the forward primer and all European fish (i.e. cytosine replacing a thymine). Tails were added at the 5′ end to be complementary with Illumina Nextera index primers. DNA amplifications were performed with 12 replicates in a final volume of 10 μL. The amplification mixture contained 1X Phusion Green Hot Start II High-Fidelity PCR Master Mix (Thermo Scientific), 0.4 μM of each of the tailed primers, 0.8 μg/μL bovine serum albumin (BSA—Thermo Scientific), 3% of Dimethyl Sulfoxide (DMSO) (Thermo Scientific), 1.5 mM of MgCl_2_ (Invitrogen), and topped up with PCR grade water (Thermo Scientific). PCR conditions consisted of an initial denaturation at 98 °C for 3 min, followed by 45 cycles of 20 s at 98 °C, 15 s at 69 °C, and 15 s at 72 °C, and a final elongation step at 72 °C for 5 min. All PCRs were performed in the presence of both a negative and positive control (i.e. a mock community with a known composition of non-native fish species). Amplification success at each step was determined by gel electrophoresis. All PCRs replicates per sample were pooled and purified using MagBind TotalPure NGS (Omega Biotek) magnetic beads with a ratio 0.8:1 (beads:DNA) to remove primer dimers.

The purified amplicons were indexed in a second PCR with a final volume of 20 μL. The PCR conditions followed Illumina’s 16S Metagenomic Sequencing Library Preparation protocol and contained 1X Phusion Green Hot Start II High-Fidelity PCR Master Mix (Thermo Scientific), 2 μL of Nextera XT i7 Index Primer (Illumina), 2 μL of Nextera XT i5 Index Primer (Illumina), 4 μL of PCR grade water (Thermo Scientific), and 2 μL of purified first-round PCR product. The second-round PCR products were purified using Mag-Bind TotalPure NGS (OMEGA BIOTEK) magnetic beads with a ratio 1:1 (beads:DNA). Purified index PCRs were quantified using a Qubit dsDNA HS Assay Kit and sized using a TapeStation D1000 ScreenTape System (Agilent), and normalized to 4 nM. The libraries were pooled in equimolar concentrations and sequencing on an Illumina MiSeq with a V2 2 × 250 bp kit, the final library was loaded at 15 pM with a 10% PhiX control spike.

### Bioinformatics

Samples were demultiplexed based on the combination of the i5 and i7 index tags. Paired-end reads for each sample were merged with USEARCH with a minimum overlap of 20% of the total read length. Forward and reverse primers were trimmed from the merged sequences using cutadapt^33^ and retained if the trimmed length was between 140 and 200 bp. These sequences were quality filtered with USEARCH to retain only those with an expected error rate per base of 0.05 or below and dereplicated by sample, retaining singletons. Unique reads from all samples were denoised in a single analysis with UNOISE, requiring retained ZOTU`s (zero-radius OTU's) to have a minimum abundance of 8 in at least one sample. A taxon-by-sample table was generated by mapping all dereplicated reads for each sample to the ZOTU representative sequences with USEARCH at an identity threshold of 97%. ZOTU's were identified via BLAST searches of the representative sequences against the nt database and a local curated database of 12S fish sequences. Identifications were based on the highest available percentage identity at 98–100%, with an e-score of 1e-20 and a hit length of at least 80% of the query sequence. In cases where multiple reference sequences match equally to the query sequence then a more conservative higher taxonomic classification is given. Only sequences with species- or genus-level identifications were included in the final results. Where a species is represented by multiple ZOTUs, the one with the highest percentage match to that species is taken as the representative. Typically, the other sequences have the same occurrence pattern and the lower sequence similarity can be attributed to PCR or sequencing errors.

## Supplementary information

Supplementary Information 1.

Supplementary Information 2.

Supplementary Information 3.

## Data Availability

The raw sequences generated during the current study are available from the corresponding author on reasonable request.
