# Supplementary material for: Aquatic suspended particulate matter as source of eDNA for fish metabarcoding
Source: Sci Rep. 2020 Sep 1;10:14352. doi: 10.1038/s41598-020-71238-w (PMC7463230; doi:10.1038/s41598-020-71238-w)
Supplement: Supplementary file 1 — Supplementary Information 1. [file 41598_2020_71238_MOESM1_ESM.pdf]

Aquatic Suspended Particulate Matter as Source of eDNA for Fish Metabarcoding

Cecilia Díaz[1*], Franziska-Frederike Wege[1], Cuong Q. Tang[2], Alexandra Crampton-Platt[2], Heinz Rüdel[1], Elke Eilebrecht[1], Jan Koschorreck[3]

[1] Fraunhofer IME, Department of Ecotoxicology, Auf dem Aberg 1, 57392 Schmallenberg, Germany

[2] Nature Metrics, CABI Site, Bakeham Lane, Egham, Surrey, UK.

[3] Federal Environment Agency (UBA), Bismarckplatz 1, 14193 Berlin, Germany

*corresponding author: cecilia.diaz@ime.fraunhofer.de

First metabarcoding results – Zebrafish crossed contamination

There was a significant number of Zebrafish sequences in all samples resulting from cross-contamination during the DNA extraction process (Table 1). Excluding the Zebrafish, a total of 23 fish taxa belonging to 10 orders, 10 families, and 23 genera were detected. The relative proportion of the fish sequences found in each of the samples is shown in Figure 1. The average species richness was 6 and ranged from 1 - 14, with the highest diversity being detected in the sample without any Zebrafish contamination (#10). Among the most commonly detected species were the Gudgeon and Common bream, which were both detected in 7 of the samples.

Table 1. Proportion of Zebrafish sequences in the samples

Location	% Zebrafisch First sampling
Koblenz	67.9
Güdingen	97.5
Prossen	93.3
Ulm	89.9
Blankenese	23.3
Weil	43.9
Bimmen	66.0
Kelheim	39.6
Dessau	15.4

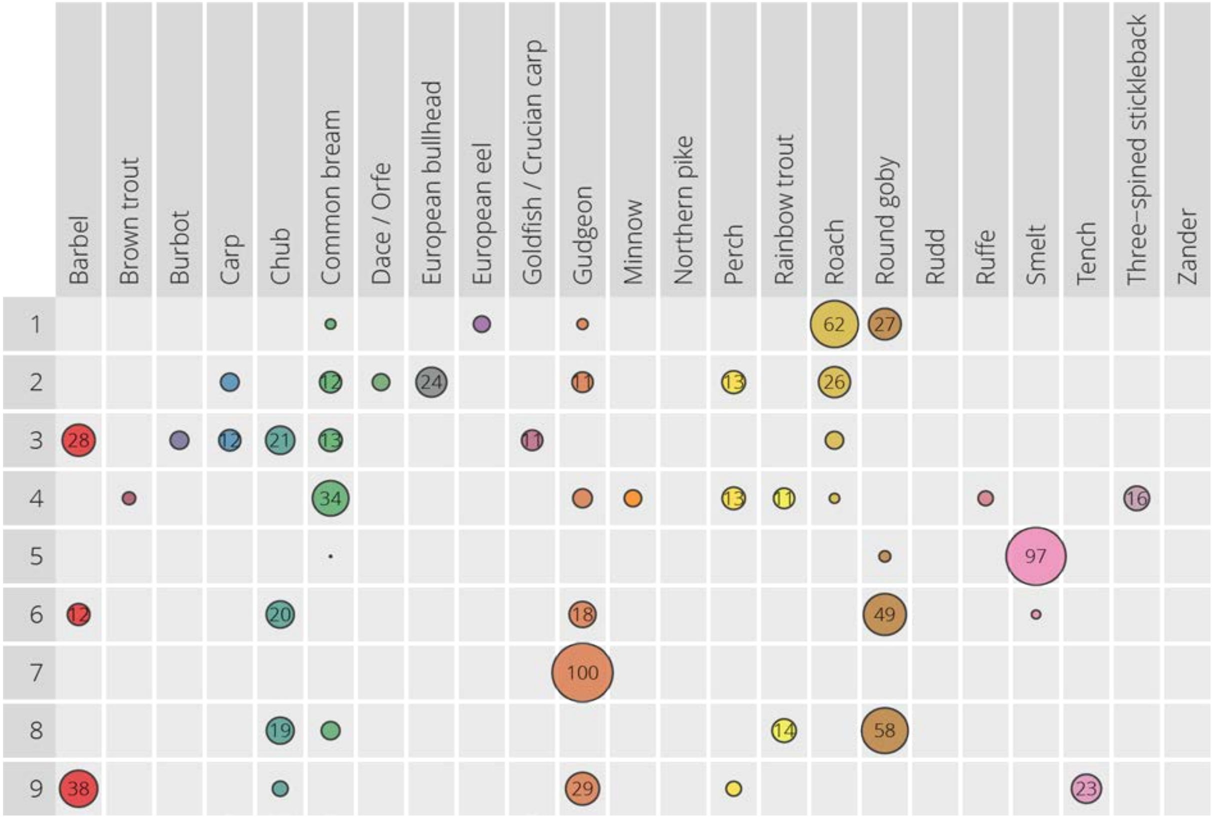

Figure 1. Proportion of the sequencing output allocated to the different species, excluding Zebrafish. 1) Koblenz, 2) Güdingen, 3) Prossen, 4) Ulm, 5) Blankenese, 6) Weil, 7) Bimmen, 8) Kehlheim, 9) Dessau
