# Supplementary material for: Aquatic suspended particulate matter as source of eDNA for fish metabarcoding
Source: Sci Rep. 2020 Sep 1;10:14352. doi: 10.1038/s41598-020-71238-w (PMC7463230; doi:10.1038/s41598-020-71238-w)
Supplement: Supplementary file 2 — Supplementary Information 2. [file 41598_2020_71238_MOESM2_ESM.pdf]

Aquatic Suspended Particulate Matter as Source of eDNA for Fish Metabarcoding

Cecilia Díaz[1*], Franziska-Frederike Wege[1], Cuong Q. Tang[2], Alexandra Crampton-Platt[2], Heinz Rüdel[1], Elke Eilebrecht[1], Jan Koschorreck[3]

[1] Fraunhofer IME, Department of Ecotoxicology, Auf dem Aberg 1, 57392 Schmallenberg, Germany

[2] Nature Metrics, CABI Site, Bakeham Lane, Egham, Surrey, UK.

[3] Federal Environment Agency (UBA), Bismarckplatz 1, 14193 Berlin, Germany

*corresponding author: cecilia.diaz@ime.fraunhofer.de

Table. List of freshwater fishes for Germany. [1, 2, 3, 4]

Order	Family	Species	FB name
Cypriniformes	Cyprinidae	Abramis brama	Common bream
Cypriniformes	Cyprinidae	Abramis sapa	White-eye bream
Acipenseriformes	Acipenseridae	Acipenser baerii baerii	Siberian sturgeon
Acipenseriformes	Acipenseridae	Acipenser gueldenstaedtii	Russian sturgeon
Acipenseriformes	Acipenseridae	Acipenser ruthenus	Sterlet
Acipenseriformes	Acipenseridae	Acipenser sturio	Sturgeon
Cypriniformes	Cyprinidae	Alburnoides bipunctatus	Spirlin
Cypriniformes	Cyprinidae	Alburnus alburnus	Bleak
Cypriniformes	Cyprinidae	Alburnus chalcoides	Danube bleak
Cypriniformes	Cyprinidae	Alburnus mento	
Clupeiformes	Clupeidae	Alosa alosa	Allis shad
Siluriformes	Ictaluridae	Ameiurus melas	Black bullhead
Siluriformes	Ictaluridae	Ameiurus nebulosus	Brown bullhead
Anguilliformes	Anguillidae	Anguilla anguilla	European eel
Cypriniformes	Cyprinidae	Aristichthys nobilis	Bighead carp
Cypriniformes	Cyprinidae	Aspius aspius	Asp
Cypriniformes	Cyprinidae	Ballerus ballerus	Zope
Cypriniformes	Balitoridae	Barbatula barbatula	Stone loach
Cypriniformes	Cyprinidae	Barbus barbus	Barbel
Cypriniformes	Cyprinidae	Blicca bjoerkna	White bream
Cypriniformes	Cyprinidae	Carassius auratus auratus	Goldfish
Cypriniformes	Cyprinidae	Carassius carassius	Crucian carp
Cypriniformes	Cyprinidae	Carassius gibelio	Prussian carp
Cypriniformes	Cyprinidae	Chondrostoma nasus	Sneep
Cypriniformes	Cobitidae	Cobitis taenia	Spined loach
Salmoniformes	Salmonidae	Coregonus albula	Vendace
Salmoniformes	Salmonidae	Coregonus arenicolus	
Salmoniformes	Salmonidae	Coregonus bavaricus	
Salmoniformes	Salmonidae	Coregonus candidus	
Salmoniformes	Salmonidae	Coregonus clupeaformis	Lake whitefish
Salmoniformes	Salmonidae	Coregonus fontanae	
Salmoniformes	Salmonidae	Coregonus gutturosus	
Salmoniformes	Salmonidae	Coregonus hoferi	
Salmoniformes	Salmonidae	Coregonus lavaretus	Common whitefish
Salmoniformes	Salmonidae	Coregonus lucinensis	
Salmoniformes	Salmonidae	Coregonus macrophthalmus	
Salmoniformes	Salmonidae	Coregonus maraena	Maraena whitefish
Salmoniformes	Salmonidae	Coregonus oxyrinchus	Houting
Salmoniformes	Salmonidae	Coregonus peled	Peled
Salmoniformes	Salmonidae	Coregonus pidschian	Humpback whitefish
Salmoniformes	Salmonidae	Coregonus renke	
Salmoniformes	Salmonidae	Coregonus wartmanni	

[1] Freyhof, J., Freshwater fish diversity in Germany, threats and species extinction. Conservation of Freshwater Fishes: Options for the Future, 2002: p. 3-22.

[2] https://fish.mongabay.com/data/Germany.htm

[3] Gaul J. (1999): Fischereibiologische Einteilung der Fließgewässer. Unter: http://www.jgaul.de/regionen.htm (Stand 22.11.2018).

[4] Internationale Kommission zum Schutz des Rheins (IKSR) (Hrsg.) (2007): Rhein-Messprogramm Biologie 2006/2007, Teil II-E. Qualitätskomponente Fische – Monitoring Rheinfischfauna (Stand 2007). Unter: https://www.iksr.org/fileadmin/user_upload/DKDM/Dokumente/Fachberichte/DE/rp_De_0173.pdf (Stand:04.04.2019)

Salmoniformes	Salmonidae	Coregonus widegreni	Valaam whitefish
Scorpaeniformes	Cottidae	Cottus gobio	Bullhead
Scorpaeniformes	Cottidae	Cottus microstomus	
Scorpaeniformes	Cottidae	Cottus perifretum	
Scorpaeniformes	Cottidae	Cottus poecilopus	Alpine bullhead
Scorpaeniformes	Cottidae	Cottus rhenanus	
Cypriniformes	Cyprinidae	Ctenopharyngodon idella	Grass carp
Cypriniformes	Cyprinidae	Cyprinus carpio carpio	Common carp
Perciformes	Moronidae	Dicentrarchus labrax	European seabass
Esociformes	Esocidae	Esox lucius	Northern pike
Petromyzontiformes	Petromyzontidae	Eudontomyzon danfordi	Carpathian lamprey
Petromyzontiformes	Petromyzontidae	Eudontomyzon vladykovi	Danubian brook lamprey
Gasterosteiformes	Gasterosteidae	Gasterosteus aculeatus aculeatus	Three-spined stickleback
Cypriniformes	Cyprinidae	Gobio gobio gobio	Gudgeon
Perciformes	Percidae	Gymnocephalus cernuus	Ruffe
Perciformes	Percidae	Gymnocephalus schraetser	Schraetzer
Salmoniformes	Salmonidae	Hucho hucho	Huchen
Cypriniformes	Cyprinidae	Hypophthalmichthys molitrix	Silver carp
Petromyzontiformes	Petromyzontidae	Lampetra fluviatilis	European river lamprey
Petromyzontiformes	Petromyzontidae	Lampetra planeri	European brook lamprey
Perciformes	Centrarchidae	Lepomis auritus	Redbreast sunfish
Perciformes	Centrarchidae	Lepomis cyanellus	Green sunfish
Perciformes	Centrarchidae	Lepomis gibbosus	Pumpkinseed
Cypriniformes	Cyprinidae	Leucaspius delineatus	Belica
Cypriniformes	Cyprinidae	Leuciscus idus	Ide
Cypriniformes	Cyprinidae	Leuciscus leuciscus	Common dace
Cypriniformes	Cyprinidae	Leuciscus souffia	Varione
Gadiformes	Lotidae	Lota lota	Burbot
Perciformes	Centrarchidae	Micropterus dolomieu	Smallmouth bass
Perciformes	Centrarchidae	Micropterus salmoides	Largemouth bass
Cypriniformes	Cobitidae	Misgurnus anguillicaudatus	Oriental weatherfish
Cypriniformes	Cobitidae	Misgurnus fossilis	Weatherfish
Perciformes	Gobiidae	Neogobius kessleri	Bighead goby
Gobiiformes	Gobiidae	Neogobius melanostomus	Round goby
Salmoniformes	Salmonidae	Oncorhynchus kisutch	Coho salmon
Salmoniformes	Salmonidae	Oncorhynchus tshawytscha	Chinook salmon
Perciformes	Cichlidae	Oreochromis niloticus niloticus	Nile tilapia
Osmeriformes	Osmeridae	Osmerus eperlanus	European smelt
Cypriniformes	Cyprinidae	Pelecus cultratus	Ziege
Perciformes	Percidae	Perca fluviatilis	European perch
Petromyzontiformes	Petromyzontidae	Petromyzon marinus	Sea lamprey
Cypriniformes	Cyprinidae	Phoxinus percnurus	Lake minnow
Cypriniformes	Cyprinidae	Phoxinus phoxinus	Eurasian minnow
Cypriniformes	Cyprinidae	Pimephales promelas	Fathead minnow

Pleuronectiformes	Pleuronectidae	Platichthys flesus	Flounder
Gobiiformes	Gobiidae	Ponticola kessleri	Kessler's goby
Perciformes	Gobiidae	Proterorhinus marmoratus	Tubenose goby
Cypriniformes	Cyprinidae	Pseudorasbora parva	Stone moroko
Gasterosteiformes	Gasterosteidae	Pungitius pungitius	Ninespine stickleback
Cypriniformes	Cyprinidae	Rhodeus sericeus	Amur bitterling
Characiformes	Characidae	Roeboides dayi	
Cypriniformes	Cyprinidae	Romanogobio albipinnatus	White-finned gudgeon
Cypriniformes	Cyprinidae	Romanogobio belingi	
Cypriniformes	Cyprinidae	Romanogobio kesslerii	Kessler's gudgeon
Cypriniformes	Cyprinidae	Romanogobio uranoscopus	Danubian longbarbel gudgeon
Cypriniformes	Cyprinidae	Rutilus meidingerii	
Cypriniformes	Cyprinidae	Rutilus pigus	
Cypriniformes	Cyprinidae	Rutilus rutilus	Roach
Salmoniformes	Salmonidae	Salmo salar	Atlantic salmon
Salmoniformes	Salmonidae	Salmo trutta fario	Brown trout
Salmoniformes	Salmonidae	Salmo trutta lacustris	
Salmoniformes	Salmonidae	Salmo trutta trutta	Sea trout
Salmoniformes	Salmonidae	Salvelinus alpinus alpinus	Charr
Salmoniformes	Salmonidae	Salvelinus evasus	
Salmoniformes	Salmonidae	Salvelinus fontinalis	Brook trout
Salmoniformes	Salmonidae	Salvelinus leucomaenis pluvius	
Salmoniformes	Salmonidae	Salvelinus namaycush	Lake trout
Salmoniformes	Salmonidae	Salvelinus profundus	
Salmoniformes	Salmonidae	Salvelinus umbla	
Perciformes	Percidae	Sander lucioperca	Pike-perch
Cypriniformes	Cyprinidae	Scardinius erythrophthalmus	Rudd
Siluriformes	Siluridae	Silurus glanis	Wels catfish
Cypriniformes	Cyprinidae	Squalius cephalus	European Chub
Salmoniformes	Salmonidae	Thymallus thymallus	Grayling
Cypriniformes	Cyprinidae	Tinca tinca	Tench
Esociformes	Umbridae	Umbra krameri	Mudminnow
Esociformes	Umbridae	Umbra pygmaea	Eastern mudminnow
Cypriniformes	Cyprinidae	Vimba vimba	Vimba
Perciformes	Percidae	Zingel streber	Danube streber
Perciformes	Percidae	Zingel zingel	Zingel
