# Supplementary material for: Aquatic suspended particulate matter as source of eDNA for fish metabarcoding
Source: Sci Rep. 2020 Sep 1;10:14352. doi: 10.1038/s41598-020-71238-w (PMC7463230; doi:10.1038/s41598-020-71238-w)
Supplement: Supplementary file 3 — Supplementary Information 3. [file 41598_2020_71238_MOESM3_ESM.pdf]

Aquatic Suspended Particulate Matter as Source of eDNA for Fish Metabarcoding

Cecilia Díaz[1*], Franziska-Frederike Wege[1], Cuong Q. Tang[2], Alexandra Crampton-Platt[2], Heinz Rüdel[1], Elke Eilebrecht[1], Jan Koschorreck[3]

[1] Fraunhofer IME, Department of Ecotoxicology, Auf dem Aberg 1, 57392 Schmallenberg, Germany

[2] Nature Metrics, CABI Site, Bakeham Lane, Egham, Surrey, UK.

[3] Federal Environment Agency (UBA), Bismarckplatz 1, 14193 Berlin, Germany

*corresponding author: cecilia.diaz@ime.fraunhofer.de

Zebrafish specific qPCR

PCR reactions were performed using Zebrafish specific Primers targeting a 81 bp long fragment (National Center for Biotechnology Information o.J.).: forward Primer 5‘-GAGAGCGTCTATAAGGAGTAC-3‘ and reverse Primer 3‘-GAGCTCATCAGAAACAGGACT-5‘ (GenBank ID XR_029715, Artuso et al. 2012).

The amplification mixture contained 10 µl of FastStart Essential DNA Green Master Mix (Roche), 1 µl of each primer (10 µM), 1 µl of the DNA template and topped up to 20 µl with PCR grade water (Roche). PCR conditions comprised of an initial denaturation at 95 °C for 3 minutes, followed 35 cycles of 5 seconds at 95 °C, 10 seconds at 60 °C and 20 seconds at 72 °C.

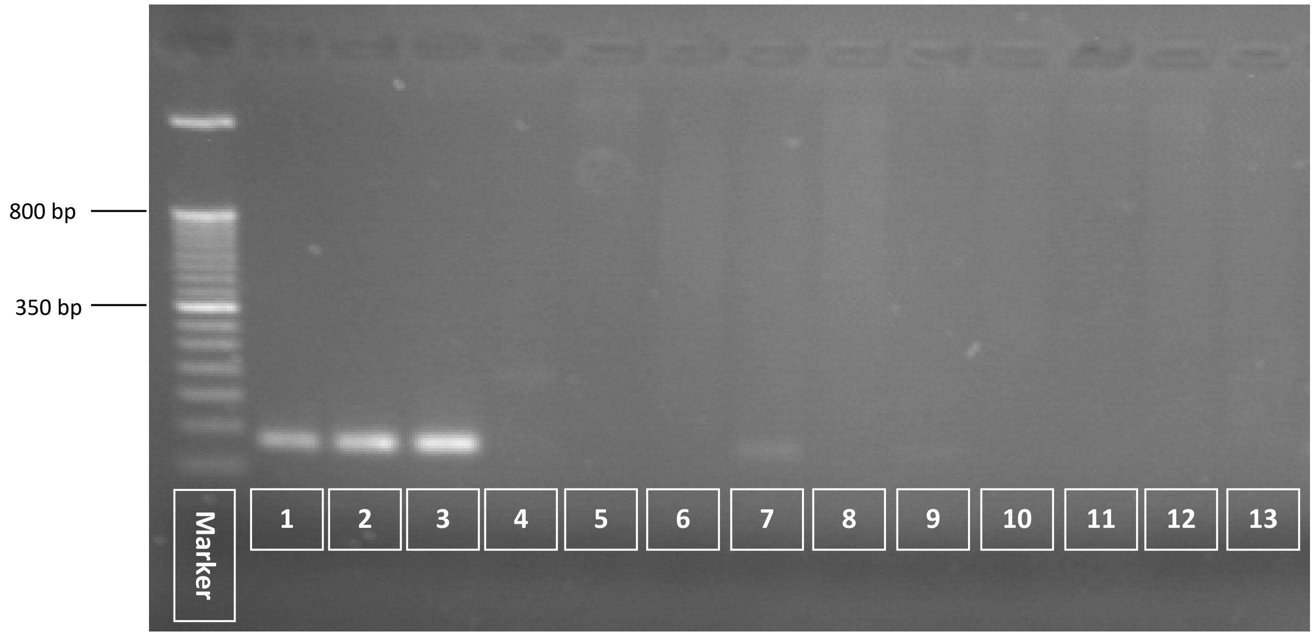

Figure 1: Detection of zebrafish DNA-contamination in SPM-samples using PCR, sampling year 2017. 1-3: positive controls –zebrafish tissue, 4: negative control – tissue from other fish species (mix without zebrafish) 5: Koblenz, 6: Güdingen, 7: Prossen, 8: Ulm, 9: Blankenese, 10: Weil, 11: Bimmen, 12: Kelheim, 13: Dessau.
